# Effects of Tea Tree Oil on B16F10 Melanoma Cells and Their Syngeneic Tumors in Mice

**DOI:** 10.3390/cimb48101014

**Published:** 2026-10-01

**Authors:** Weiwei Lei, Meng Zhang, Jiayu Xie, Yaxin Yang, Xin Chen, Yumei Zhang

**Affiliations:** 1 College of Veterinary Medicine, Yangzhou University, Yangzhou 225009, China; 2 Jiangsu Co-Innovation Center for Prevention and Control of Important Animal Infectious Diseases and Zoonoses, Yangzhou University, Yangzhou 225009, China; 3 Joint International Research Laboratory of Agriculture and Agri-Product Safety, Ministry of Education of China, Yangzhou University, Yangzhou 225009, China

**Keywords:** tea tree oil, anticancer effect, apoptosis, B16F10, syngeneic tumors

## Abstract

Tea tree oil (TTO) presents many biological and pharmacological activities in animals and humans. In this study, six TTO preparations (TTO A–F) with different terpinen-4-ol and isoterpinene contents were screened for cytotoxicity against B16F10 melanoma cells in vitro; the two most active preparations (TTO B and TTO E) were subsequently evaluated by intratumoral injection (0.1 mL of 10% TTO once daily for 7 days; five mice per group) in a syngeneic B16F10 tumor model in C57BL/6 mice, and the mechanistic analyses focused on TTO E. The results showed that 0.0125–0.1% TTOs exhibited dose-dependent cytotoxic effects in B16F10 cells, with TTO E exhibiting a stronger inhibitory effect than the others. Intratumoral injection of TTO E significantly suppressed the growth of B16F10 syngeneic tumors, with reductions in tumor volume of 69.1% and in tumor weight of 61.2% on day 14 compared with the PBS control. Apoptosis and cleavage of caspase-3/-9 in B16F10 cells in vitro and in their syngeneic tumors in mice were significantly induced by TTO E. These data indicate that TTO E shows antitumor activity towards B16F10 cells in vitro and in a syngeneic mouse model in vivo through an apoptosis-associated mechanism; this merits further preclinical evaluation.

## 1. Introduction

Tea tree oil (TTO)—a steam-distillation extract from the leaves of the tea tree (*Melaleuca alternifolia*)—contains more than 100 compounds, typically ~50% monoterpenes, ~50% monoterpene derivatives, and a small amount of sesquiterpenes [1,2]. TTO has been demonstrated to have antifungal [3,4,5], antiviral [6,7], anti-bacterial [8,9], and anti-inflammatory bioactivities [10,11]. Furthermore, terpinen-4-ol, γ-terpinene, and α-terpinene have been observed to exhibit trypanocidal effects when used individually and together, both in vitro and in vivo [12], while TTO and its main component terpinen-4-ol are effective in the treatment of demodex-related diseases [13]. Moreover, α-terpinene has been demonstrated to exhibit antioxidant activity [14,15,16].

The cytotoxic activities of several monoterpenes and sesquiterpenes against cancer cell lines in vitro and tumors in vivo have also been reported. Accordingly, TTO exhibits antitumor effects on human and mouse breast cancer MCF-7 and 4T1 cells [17]; human A-375 malignant melanoma cells; wild-type and adriamycin-resistant M14 cells [18,19]; HEp-2 squamous cell carcinoma cells [18]; and murine AE17 mesothelioma cells (and their mouse implantation tumors) in vitro and in vivo [20]. Furthermore, terpinen-4-ol has been reported to inhibit the growth of colorectal, pancreatic, prostate, and gastric cancer cells in a dose-dependent manner as well as having significant enhancement effects on several chemotherapeutic and biological anticancer agents [21]. Finally, terpinen-4-ol induces apoptosis in human non-small cell lung cancer [22] and HCT116 and RKO colorectal cancer cell lines through reactive oxygen species generation [23]. In summary, the characteristic TTO components terpinen-4-ol, α-terpineol, γ-terpinene, terpinolene, and 1,8-cineole exhibit different antibiosis, anti-inflammation, antioxidation, and antitumor effects with different mechanisms of action. Furthermore, they exhibit some synergistic effects with each other and with other chemotherapy drugs [10,24,25].

Terpinen-4-ol has been shown to exhibit antitumor, antifungal, antibiosis, and anti-Trypanosoma evansi effects, but little information on isoterpinene has been reported. The present study was therefore designed to (i) compare the in vitro cytotoxicity of six TTO preparations (labeled A–F) with distinct terpinen-4-ol/isoterpinene profiles against B16F10 melanoma cells, (ii) evaluate the in vivo antitumor activity of the two most active preparations in a syngeneic B16F10/C57BL/6 model following intratumoral administration, and (iii) explore the apoptosis-associated mechanism involved. The B16F10/C57BL/6 model was selected because it is an immunocompetent, syngeneic melanoma model that allows antitumor activity to be assessed in the presence of a functional immune system, whereas previous TTO studies have mainly used human melanoma, breast, lung, and mesothelioma models. Unlike earlier studies that tested a single standardized TTO or isolated terpinen-4-ol, the parallel comparison of six chemically distinct TTO preparations provides an initial, composition-based perspective on how differences in terpinen-4-ol and isoterpinene content relate to antitumor activity, and therefore helps to determine their potential as anticancer agents.

## 2. Materials and Methods

### 2.1. TTO Samples

Six TTOs were obtained from commercial essential oils of the tea tree (*Melaleuca alternifolia*) leaves using different extraction processes by Wuxi Chenfang Tech Co., Wuxi, China. The compositions of the TTOs are shown in Table 1. The chemical compositions of the oils (Table 1) were determined and provided by the supplier. The oils were stored in sealed amber-glass bottles at 4 °C and protected from light until use.

The TTO samples were dissolved in DMSO and serially diluted with culture medium to obtain concentrations ranging from 0.00625% to 0.1% (*v*/*v*). The final DMSO concentration was 0.05% (*v*/*v*) in all treatment groups and in the corresponding vehicle control.

### 2.2. Cell Culture

B16F10 melanoma cells were purchased frozen in liquid nitrogen from the American Type Culture Collection (ATCC) (Manassas, VA, USA). They were cultured in Dulbecco’s modified Eagle’s medium (DMEM) supplemented with 5% fetal bovine serum (FBS) and 1% penicillin/streptomycin in an incubator containing 5% CO_2_ at 37 °C.

### 2.3. Cell Viability Assay

The cells (1 × 10^4^ cells/mL) were seeded in 96-well plates (200 µL/well) in complete medium at 5% CO_2_ and 37 °C overnight. The TTOs were added to the cells at different concentrations. The MTT assay was used to assess TTO-induced changes in cellular metabolic activity as a surrogate measure of cell viability/cytotoxicity rather than cell proliferation. PBS and 0.05% (*v*/*v*) DMSO vehicle controls were included in each experiment. After 24 h, the medium was replaced with fresh media containing 1 mg/mL 3-(4,5-dimethylthiazol-2-yl)-2,5-diphenyltetrazolium bromide (MTT), and the plate was incubated for 2 h. The absorbances of the samples were measured at 570 nm using an ELX-800 ELISA reader (BioTek, Winooski, VT, USA). To quantify the cytotoxic potency, the half-maximal inhibitory concentration (IC50) values were calculated by nonlinear regression (log(inhibitor) vs. normalized response) using GraphPad Prism. The experiments were performed in triplicate.

### 2.4. Flow Cytometry for Apoptosis

The cells (1 × 10^5^ cells/mL) were seeded in 6-well plates (2 mL each well) for 24 h and then exposed to different doses of TTO E for 24 h. Cells were harvested, washed, and resuspended in 500 µL of binding buffer. Cell suspensions were then incubated with 5 µL of annexin V-FITC and 5 µL of PI for 10 min at room temperature in the dark. The cells were then analyzed using a FACSCalibur flow cytometer (BD Biosciences, Franklin Lakes, NJ, USA). Cells were first gated by forward/side scatter (FSC/SSC) to exclude debris, and fluorescence compensation was performed using single-stained annexin V-FITC and PI controls. Apoptotic cells were defined as annexin V-positive events (early apoptosis: annexin V+/PI−; late apoptosis/secondary necrosis: annexin V+/PI+), and the percentages of apoptotic cells were quantified using FlowJo software (version 7.6, BD Biosciences).

### 2.5. Mouse Syngeneic Tumor Model for Measuring Antitumor Activity In Vivo

C57BL/6 mice (female, 6–8 weeks old, 18–20 g) were obtained from the Center of Comparative Medicine, Yangzhou University (Yangzhou, China). All mice were group-housed (5 mice per cage) in individually ventilated cages under specific-pathogen-free (SPF) conditions and allowed ad libitum access to a standard rodent chow diet and drinking water. The environmental conditions were maintained at 22 ± 2 °C and a relative humidity of 55 ± 5% under a 12 h light/12 h dark cycle. All mice were acclimatized to these housing conditions for one week prior to experimentation. Mice were randomly assigned to experimental groups. The experimenters were not blinded to group allocation during treatment but were blinded during histopathological analysis and data measurement. Humane endpoints were defined as tumor diameter exceeding 1.5 cm, body weight loss exceeding 20%, or signs of severe distress; no animals reached these endpoints during the study.

The B16F10 cells were harvested and resuspended at a concentration of 1 × 10^7^ cells/mL in PBS, and 0.2 mL was injected subcutaneously into the right flanks of the mice (day 0). When the tumors became palpable, the mice were randomly divided into four groups of five mice each (20 mice in total): a PBS vehicle-control group, a TTO B group, a TTO E group, and a positive-control group receiving cyclophosphamide (Cy, 1.2 mg/mL). Because of the hydrophobic nature of the oil, the 10% (*v*/*v*) TTO formulations for intratumoral administration were prepared as emulsions in PBS containing 0.05% (*v*/*v*) DMSO; the PBS control group received the same vehicle formulation and thus served as a vehicle-matched control. Intratumoral injections (0.1 mL per mouse per day, corresponding to approximately 10 mg of TTO per injection) were performed once daily for 7 days, and the mice were followed up until day 14, when tissue samples were collected.

During the experiment, the body weights and food and water intakes of the animals were monitored daily. The length (L) and width (W) of the tumors were obtained once a day using vernier calipers, and the tumor volume was calculated using the formula 1/2 L × W^2^. On day 14, the mice were anesthetized by intraperitoneal injection of pentobarbital sodium and sacrificed by cervical dislocation, and the tumors were excised. The tumors were then weighed, fixed overnight in neutral buffered formalin, embedded in paraffin, and sectioned. Then, hematoxylin and eosin (HE) staining and immunohistochemical analysis for cleaved caspase-3 were performed. The tumor paraffin sections were also analyzed using a TUNEL assay kit-HRP-DAB (ab206386, Abcam, Cambridge, UK) according to the manufacturer’s instructions. Images were obtained using a Leica light microscope (Leica, Wetzlar, Germany) at 200× magnification.

### 2.6. Western Blot Analysis

Cells and tumor tissues were lysed in ice-cold RIPA lysis buffer with phenylmethyl sulfonyl fluoride and a phosphatase inhibitor cocktail (Beyotime, Shanghai, China). Equal amounts of protein (30 µg per lane) were separated using 8% sodium dodecyl sulfate-polyacrylamide gel electrophoresis and transferred to 0.45 µm nitrocellulose membranes. After blocking in 5% skimmed milk for 2 h at room temperature, membranes were incubated overnight at 4 °C with the primary antibodies against cleaved caspase-3 and -9 (No. 9661, No. 9501, Cell Signaling Technology, Danvers, MA, USA) at 1:500 dilution, and β-actin (No. BA2305, BOSTER Biological Technology Co., Ltd., Wuhan, China) at 1:250 dilution. Blots were developed using an enhanced chemiluminescence detection system (Bio-Rad Laboratories). The intensities of the blots were measured using computer-assisted densitometric analysis (GS-800 densitometer, Quantity One; Bio-Rad, Hercules, CA, USA). The membranes were then incubated with the corresponding HRP-conjugated secondary antibodies for 1 h at room temperature. The band intensities of the target proteins were normalized to those of β-actin. 

### 2.7. Statistical Analysis

Results are expressed as mean ± standard deviation (SD) for pooled independent experiments (at least three biological replicates for the in vitro studies; *n* = 5 animals per group for the in vivo studies, with individual animals treated as the statistical units). Statistical comparisons between the two groups were made using an unpaired two-tailed Student’s *t*-test. For multiple group comparisons, one-way ANOVA followed by Tukey’s post hoc test was used. *p* values < 0.05 were considered statistically significant (* *p* < 0.05, ** *p* < 0.01). All analyses were performed using GraphPad Prism software (version 8.0, GraphPad Software Inc., San Diego, CA, USA). The repeatedly measured longitudinal tumor-volume data were analyzed by repeated-measures two-way ANOVA (treatment × time) followed by Tukey’s post hoc test.

## 3. Results

### 3.1. Cytotoxicity Towards B16F10 Cells In Vitro

**Figure 1:**
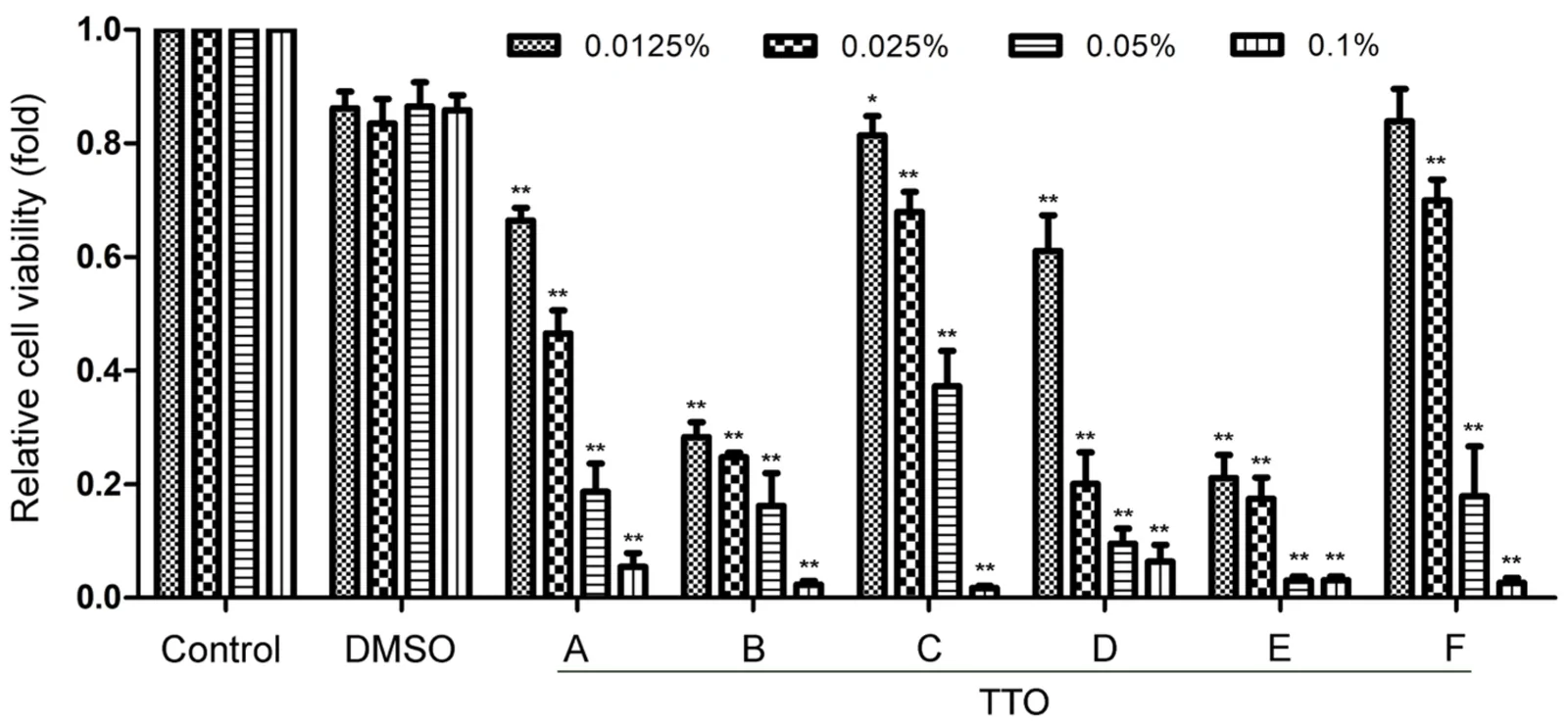
Cytotoxicities of TTOs towards B16F10 tumor cells as determined by MTT. * *p* < 0.05, ** *p* < 0.01, compared to the control. Data are means ± SD from at least three independent experiments; one-way ANOVA followed by Tukey’s post hoc test was used.

The viabilities of the B16F10 cells treated with the TTO samples were determined by MTT assay, and the results are shown in Figure 1. The relative viabilities of the B16F10 cells in the DMSO control were not significantly different from those in the PBS control. The viabilities of B16F10 cells treated with TTO samples A, B, D, and E in the concentration range 0.0125–0.1% were all significantly decreased (*p* < 0.01), whereas TTOs C and F significantly decreased the viability of B16F10 cells at 0.025–0.1% (*p* < 0.01), with ~2% viability at the 0.1% dose. The 24 h IC50 values were 0.0233%, 0.0381%, 0.0153% and 0.0327% for A, C, D and F, respectively. For B and E, cell viability decreased below 50% at 0.0125% (the lowest tested concentration), with IC_50_ < 0.0125% (Table S1). Based on the IC50 values and the overall dose–response curves, TTOs B and E showed the lowest IC50 values, followed by TTOs A and D, while TTOs C and F were the least potent.

### 3.2. Growth Suppression of B16F10 Syngeneic Tumors in Mice

**Figure 2:**
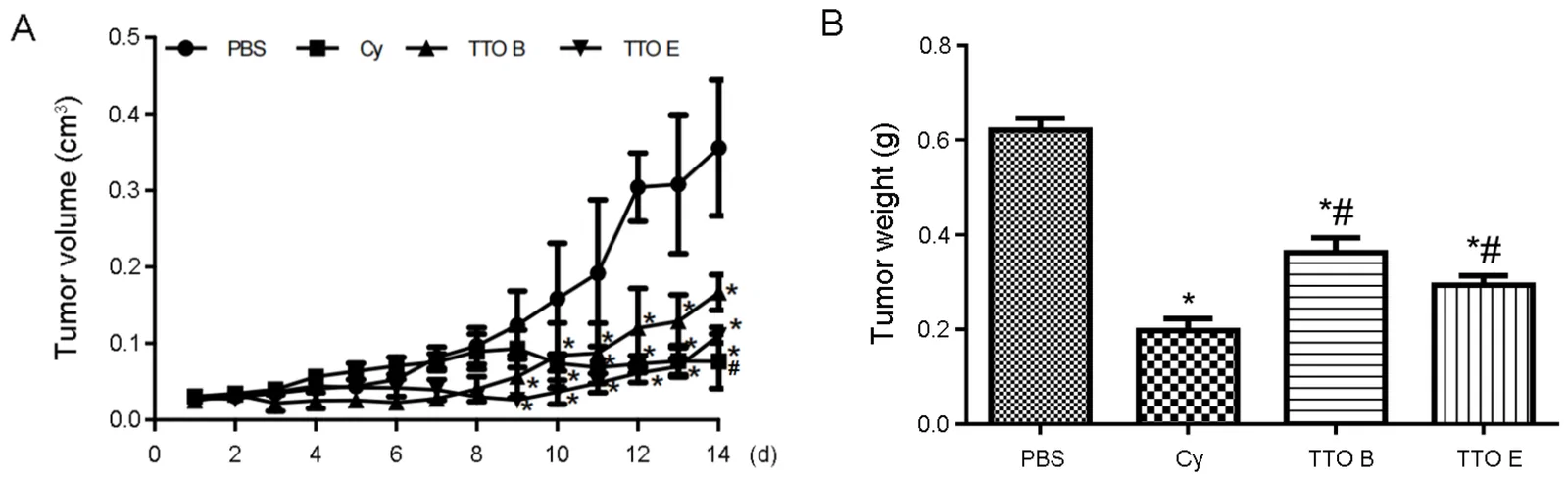
Effects of the different TTO treatments by intratumoral injection on B16F10 syngeneic tumors in mice (*n* = 5 mice per group). (**A**) Tumor volumes (cm^3^; mean ± SD) measured daily from day 1 to day 14, analyzed by repeated-measures two-way ANOVA followed by Tukey’s post hoc test. (**B**) Tumor weights (g) on day 14, analyzed by one-way ANOVA followed by Tukey’s post hoc test. * *p* < 0.05, compared to the control; # *p* < 0.05, compared to the cyclophosphamide (Cy) group.

The effects of the different treatments on the growth of the B16F10 syngeneic tumors are shown in Figure 2. As shown in Figure 2A, the tumors were visible throughout the entire experiment. The tumor volumes in the PBS control and TTO B groups gradually increased, while that in the cyclophosphamide group increased before day 9 and slowly decreased thereafter. The tumors in the TTO B, TTO E, and cyclophosphamide groups were all smaller than those in the PBS control, with significant differences being observed from day 9 onwards (*p* < 0.01). Additionally, the TTO B and the cyclophosphamide group were also statistically significant on day 14 (*p* < 0.05). Compared with the PBS group, distinct decreases in tumor volume were observed on day 14 after the administration of TTO B, TTO E, and cyclophosphamide (53.1%, 69.1%, and 78.7%, respectively).

The tumor weights (Figure 2B) in the TTO B, TTO E, and cyclophosphamide groups were significantly lower than those in the PBS control (*p* < 0.05). Considerable reductions in tumor weight were observed for TTOs B and E, being 41.5% and 61.2%, respectively. The tumors in the TTO B and TTO E groups were significantly heavier than those in the cyclophosphamide group (*p* < 0.05). No significant differences in body weight or in food and water intake were observed between the TTO-treated groups and the PBS control during the study period, suggesting that intratumoral TTO administration produced no obvious systemic toxicity at the dose used.

### 3.3. Apoptosis in B16F10 Cells In Vitro

Based on the in vitro cytotoxicity results (Figure 1; Table S1) and the in vivo antitumor activity (Figure 2), TTO B and TTO E were the two most active preparations and were therefore selected for the animal study. Because TTO E showed the strongest overall activity in both the in vitro and in vivo settings, and in order to limit the number of animals and analyses performed, the subsequent apoptosis and caspase-activation analyses focused on TTO E.

**Figure 3:**
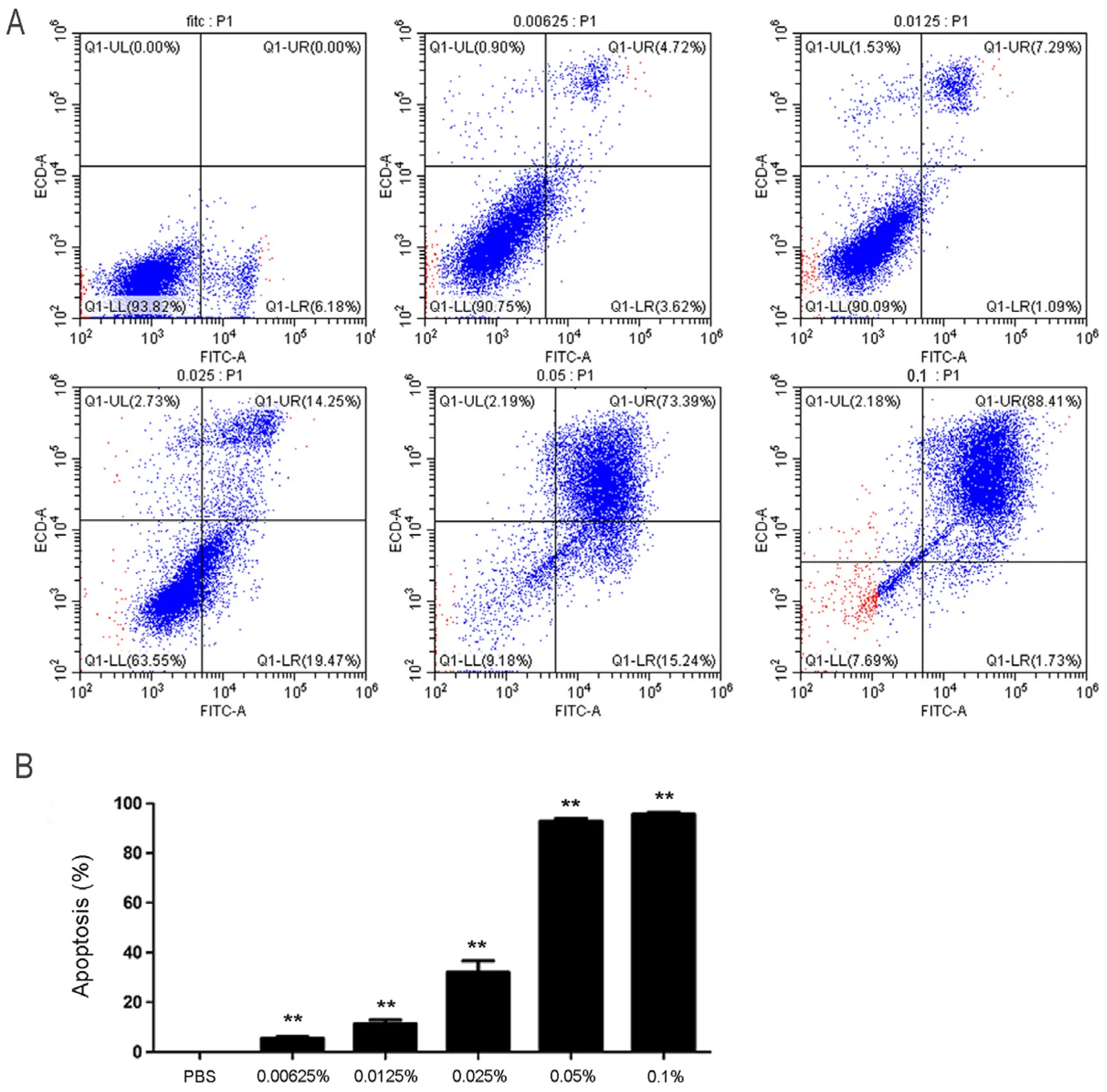
Effects of TTO on cell apoptosis in B16F10 cells. (**A**) Flow cytometry analysis of TTO E-induced apoptosis in B16F10 cells. (**B**) Histograms showing percentage apoptosis for each treatment. Data are means ± SD of three independent experiments; one-way ANOVA followed by Tukey’s post hoc test was used. ** *p* < 0.01 compared with that of the PBS control.

The effect of TTO E on apoptosis in B16F10 cells in vitro is shown in Figure 3. TTO E at concentrations in the range 0.00625–0.1% induced significantly more apoptosis in B16F10 cells compared to the control (*p* < 0.01), and the increase is dose-dependent. The apoptosis of B16F10 cells induced by 0.00625–0.025% TTO E was 8–32%. TTO E at 0.05% and 0.1% induced more than 85% cell apoptosis.

### 3.4. Apoptosis in B16F10 Syngeneic Tumors in Mice

**Figure 4:**
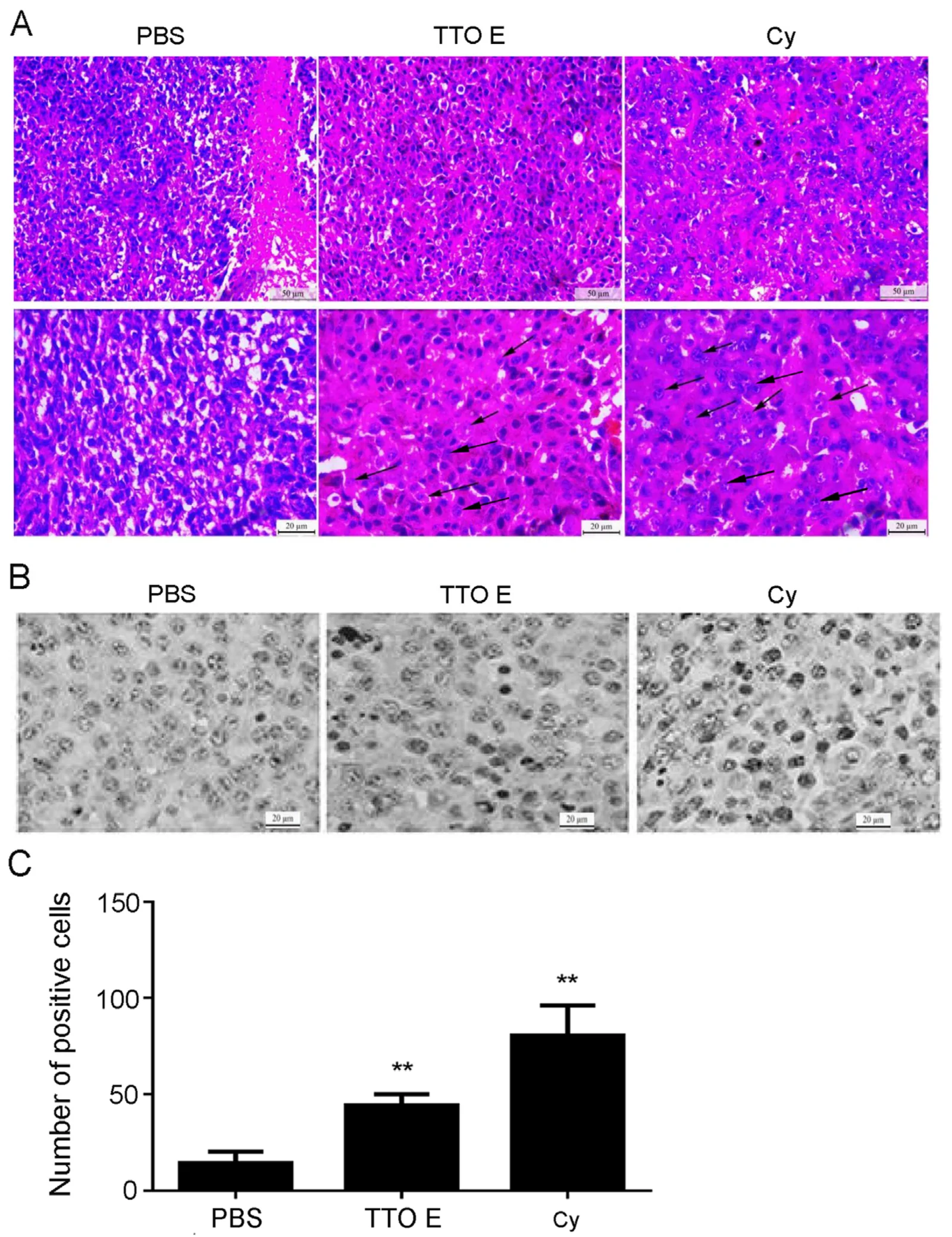
Effects of TTO E on apoptosis in B16F10 syngeneic tumors in mice. (**A**) HE staining analysis of tumors at different magnifications; arrows indicate apoptotic cells with condensed cytoplasm and hyperchromatic nuclei, and arrowheads indicate nuclear fragmentation. (**B**) TUNEL staining of tumor tissues; arrows indicate TUNEL-positive cells. (**C**) Quantification of TUNEL-positive cells in five random visual fields per tumor (five tumors per group, one tumor per mouse). Data are means ± SD; ** *p* < 0.01 compared with that of the PBS control. (one-way ANOVA followed by Tukey’s post hoc test).

HE staining analysis results for the tumors from the B16F10 syngeneic tumor model are shown in Figure 4A. The cell density in the tumors from the PBS control was clearly higher than that in the TTO E and cyclophosphamide groups. Apoptotic cells with condensed cytoplasm and condensed, hyperchromatic chromatin (arrows) as well as nuclear fragmentation (arrowheads) were clearly observed in the TTO E and cyclophosphamide groups.

For TUNEL analysis, as shown in Figure 4B/C, the numbers of positively stained cells in the tumors treated with TTO E and cyclophosphamide are significantly higher than that for the control, and the number of positive cells for cyclophosphamide is approximately double that for TTO E.

### 3.5. Caspase-3/-9 Activation in B16F10 Cells and Their Syngeneic Tumors in Mice

**Figure 5:**
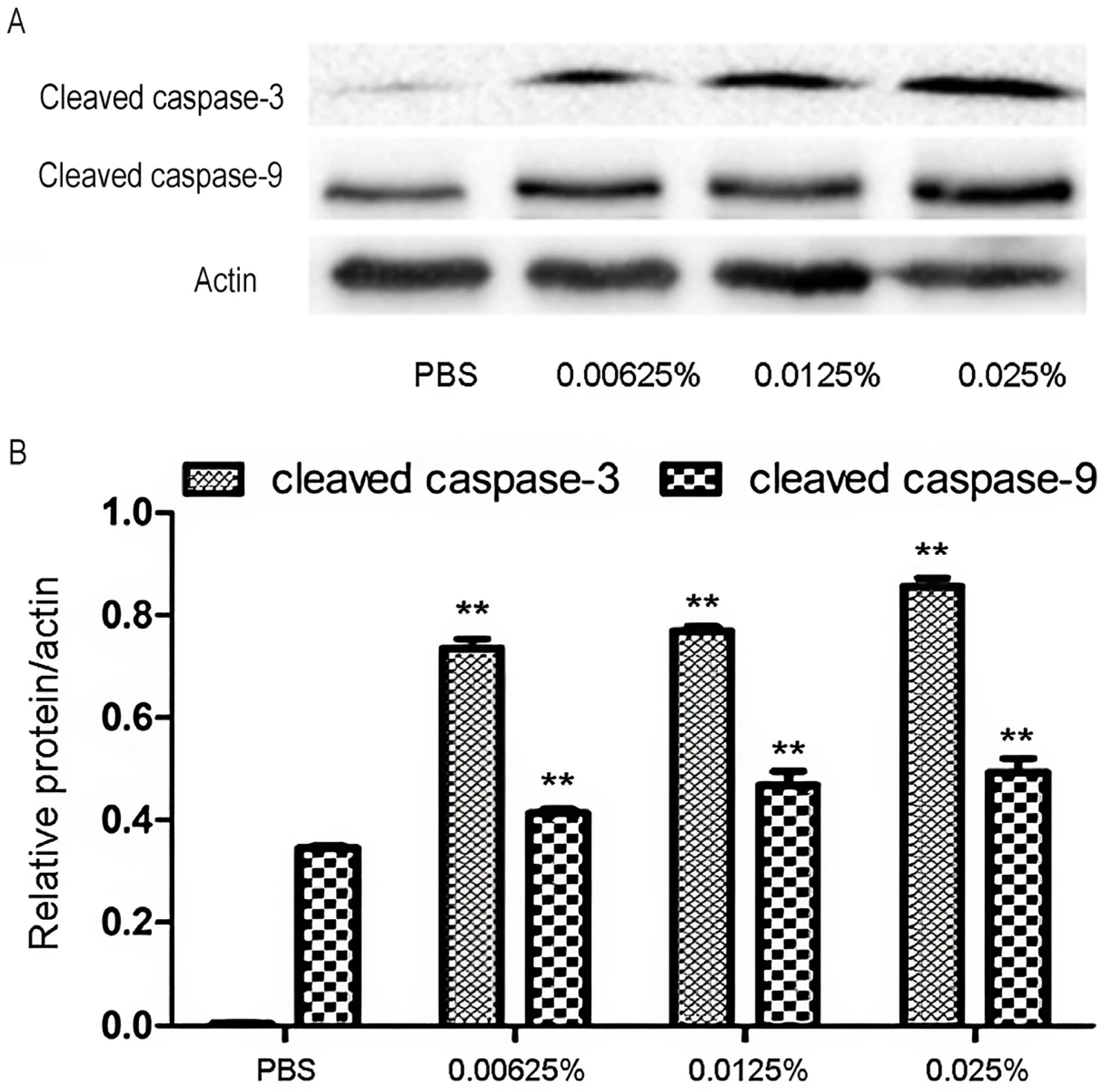
Effects of TTO E on caspase-3/-9 expression in B16F10 cells. (**A**) Western blot analysis results for B16F10 cells treated with the indicated concentrations of TTO E for 24 h. (**B**) Densitometric quantification of the bands normalized to β-actin. Data are means ± SD of three independent experiments; ** *p* < 0.01 compared with that of the PBS control (one-way ANOVA followed by Tukey’s post hoc test).

The effects of TTO E on the expression of the apoptosis-related proteins caspase-3 and caspase-9 in B16F10 cells are shown in Figure 5. The expression of cleaved caspase-3 and cleaved caspase-9 in B16F10 cells treated with 0.00625–0.025% TTO E in vitro was significantly higher than that in the control (*p* < 0.01). The level of cleaved caspase-9 in the PBS control is ~0.36 and those for 0.00625–0.025% TTO E treatment increased 1.21–1.42-fold. Furthermore, cleaved caspase-3 was not detected for the PBS control, and for the TTO E treatment it is clearly higher.

**Figure 6:**
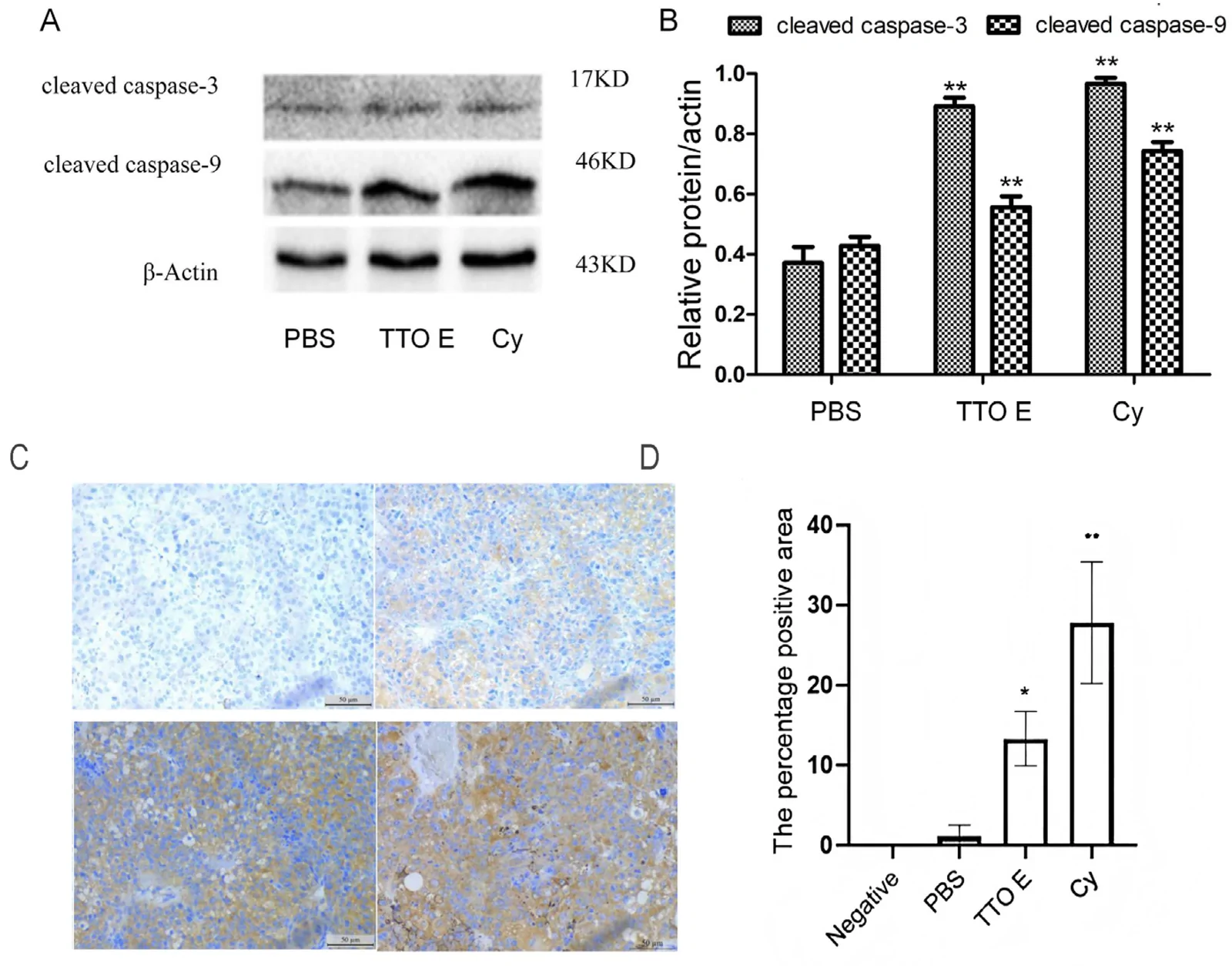
Effects of TTO E on caspase-3/-9 expression in B16F10 syngeneic tumors in mice. (**A**) Western blot analysis results for the cleaved forms of caspase-9 and caspase-3 in the tissue lysates of tumors collected on day 14; each lane represents an individual tumor from a separate mouse. (**B**) Densitometric quantification of the bands normalized to β-actin. (**C**) Immunohistochemical analysis results for cleaved caspase-3 in the B16F10 tumors; the negative control was incubated with PBS instead of the primary antibody. (**D**) The percentage of positive area of cleaved caspase-3 staining. Data are means ± SD; *n* = 5 mice per group, with five random visual fields evaluated per tumor; * *p* < 0.05, ** *p* < 0.01 compared with that of the PBS control.

The effects of TTO E on caspase-3/-9 expression in the B16F10 syngeneic tumors are shown in Figure 6. The levels of cleaved caspase-3/-9 in the tumors treated with TTO E and cyclophosphamide were all significantly increased (*p* < 0.01). Compared with the PBS control, increases of 2.19- and 2.35-fold for cleaved caspase-3 and 1.37- and 1.84-fold for cleaved caspase-9 were observed in the TTO E and cyclophosphamide groups, respectively (Figure 6B).

The results of the immunohistochemical analysis for cleaved caspase-3 in the tumors are shown in Figure 6C,D. The positive cleaved caspase-3 staining in the PBS control tumors was clearly weaker than that in the TTO E and cyclophosphamide groups; the percentages of positive area were 1.1%, 9.3% and 27.8%, respectively (*p* < 0.01).

## 4. Discussion

Previous studies have demonstrated the in vitro and in vivo anticancer efficacies of TTO and its major component terpinen-4-ol for a range of tumors, including skin cancer [20,21,26,27,28], lung cancer [23,27], gastrointestinal cancer [24], and breast cancer [18]. However, relatively little information on the effect of isoterpinene is available.

In our present investigation, the different TTOs all demonstrated anticancer activity to varying degrees. For example, the inhibition of B16F10 cells in vitro was not identical for the six TTO samples: TTOs B and E were superior to TTOs A and D, which were in turn superior to TTOs C and F. Upon comprehensive analysis of the results of the in vitro and in vivo experiments, TTOs B and E appeared to inhibit B16F10 tumor cells more strongly than the other four preparations, as reflected by their lower 24 h IC50 values (Table S1), the near-complete loss of cell viability at 0.1% (*v*/*v*), and the in vivo reductions in tumor volume (53.1% and 69.1%) and tumor weight (41.5% and 61.2%) compared with the PBS control (*p* < 0.01).

The inhibition of B16F10 tumor cells by the TTOs did not show a simple relationship with their concentrations of terpinen-4-ol or isoterpinene. For instance, the higher concentrations of terpinen-4-ol in TTOs A and C did not lead to stronger inhibition, while a lower concentration of terpinen-4-ol combined with a higher isoterpinene concentration (TTO D) was associated with stronger inhibition. However, the maximum isoterpinene concentration was found in TTO F, which showed a comparatively weaker inhibitory effect. It must be emphasized that these observations are descriptive: because the six oils are complex mixtures in which several constituents vary simultaneously, the present study design cannot establish which constituent, or combination of constituents, caused the observed differences in activity. Accordingly, a combined action of terpinen-4-ol and isoterpinene should be regarded as a hypothesis arising from the compositional patterns rather than a demonstrated synergistic effect. This hypothesis will be tested in future studies using isolated compounds and formal drug-interaction analysis (e.g., the Chou–Talalay method) to identify the percentages of terpinen-4-ol and isoterpinene in TTO that provide the maximum inhibition of B16F10 tumors.

We found that TTO induces B16F10 tumor cell apoptosis in vitro and in vivo using flow cytometry, HE and TUNEL staining, Western blot, and immunohistochemical analyses. The cell apoptosis induced by TTO is clearly higher than that of the control according to the results of Annexin V/PI, HE and TUNEL staining. A previous study has demonstrated that caspase-9 is involved in the original phase of apoptosis whereas caspase-3 is involved in the execution phase of apoptosis [29]. The expression of the apoptosis-related proteins cleaved caspase-3 and -9 are significantly increased upon TTO treatment compared with the control according to Western blot and immunohistochemical analyses.

The apoptosis induced by TTO in the present study is similar to that observed in other studies. Terpinen-4-ol has been shown to induce caspase-dependent apoptosis in human melanoma cells. TTO or terpinen-4-ol has been reported to induce apoptosis in A-375 and HEp-2 cancer cell lines [19], MCF-7 cells [18], and human leukemic HL-60 cells [30]. However, terpinen-4-ol has also been shown to induce necrosis in AE17 murine mesothelioma and B16 murine melanoma cells [26]. Terpinen-4-ol induces apoptosis in human non-small cell lung cancer cells A549 and CL1-0 via activated forms of caspase-9 and caspase-3 as well as PARP cleavage, in which terpinen-4-ol-elicited apoptosis is p53 dependent by RNA interference [23]. In addition, TTO and terpinen-4-ol have recently been shown to synergistically enhance the efficacy of dabrafenib/trametinib targeted therapy in melanoma models through apoptosis induction [31], and the anticancer properties of terpinen-4-ol, including its effects on mitochondrial apoptotic signaling, have been comprehensively reviewed [32]. In summary, the anticancer mechanisms of TTO and/or terpinen-4-ol on various tumors involve autophagic and apoptotic cell death, necrosis, cell cycle arrest, and reactive oxygen species [24,31,32].

This study has several limitations that should be acknowledged. First, only a single tumor cell line (B16F10) was used. Second, the animal groups were small (*n* = 5 per group), and the experimenters were not blinded to group allocation during the in vivo treatment administration (histopathological analysis was blinded). Third, the follow-up period was short (14 days). Fourth, TTO was delivered by intratumoral injection, which bypasses systemic pharmacokinetic barriers and therefore does not model clinical systemic administration. Finally, only whole oils rather than isolated constituents were tested, which precludes attribution of the observed effects to individual components. In addition, the chemical compositions of the oils were not independently characterized in our laboratory, as the compositional data were provided by the supplier. These limitations should be considered when interpreting the present findings.

## 5. Conclusions

In summary, selected TTO preparations, particularly TTO E, reduced the viability of B16F10 melanoma cells in vitro in a dose-dependent manner and suppressed the growth of syngeneic B16F10 tumors in C57BL/6 mice following intratumoral administration, with the antitumor effect being associated with caspase-3/-9 activation and increased apoptosis. The respective contributions of terpinen-4-ol, isoterpinene, and the other oil constituents to this activity remain unresolved. Further work—including the use of isolated constituents and formal interaction analysis, the development of suitable formulations for systemic administration, and evaluation in additional melanoma models—is required before the therapeutic applicability of TTO in melanoma can be established.

## Figures and Tables

**Table 1:** Compositional details of the TTOs considered in this study (%, *v*/*v*).

	Terpinen-4-ol	Isoterpinene	α/γ-Terpinene	1,8-Cineole	Others
A	69.4	11.7	11.2	3.5	4.2
B	71.6	12.5	10.6	3.6	1.7
C	63.0	19.5	9.1	2.5	5.9
D	52.1	28.4	11.6	3.6	4.3
E	68.0	10.0	13.3	3.4	5.3
F	14.4	70.0	11.5	2.7	1.4

## Data Availability

The raw data supporting the conclusions of this article will be made available by the authors on request.

## Supplementary Materials

The following supporting information can be downloaded at: https://www.mdpi.com/article/10.3390/cimb48101014/s1.

## Abbreviations

The following abbreviations are used in this manuscript:

TTO	Tea tree oil
MTT	3-(4,5-dimethylthiazol-2-yl)-2,5-diphenyltetrazolium bromide
DMEM	Dulbecco’s modified Eagle’s medium (DMEM)
Cy	Cyclophosphamide
TUNEL	TdT-mediated dUTP Nick-End Labeling
TTO B	Tea tree oil B
TTO E	Tea tree oil E
DMSO	dimethyl sulfoxide
PBS	phosphate-buffered saline
