# Supplementary material for: Effects of Tea Tree Oil on B16F10 Melanoma Cells and Their Syngeneic Tumors in Mice
Source: Curr Issues Mol Biol. 2026 Oct 1;48(10):1014. doi: 10.3390/cimb48101014 (PMC13703512; doi:10.3390/cimb48101014)
Supplement: Supplementary file 1 [file cimb-48-01014-s001.zip › cimb-4568206-supplementary.pdf]

Table S1. Cytotoxicity of TTO samples A–F (IC50 values)		
**Sample**	**IC50 (%)**	**Note**
A	0.0233	Calculated by nonlinear regression
B	< 0.0125	Viability <50% at lowest tested concentration
C	0.0381	Calculated by nonlinear regression
D	0.0153	Calculated by nonlinear regression
E	< 0.0125	Viability <50% at lowest tested concentration
F	0.0327	Calculated by nonlinear regression
